# The Phase 3 Study ECHELON-1 Evaluating Brentuximab Vedotin in Patients With Newly Diagnosed Hodgkin Lymphoma Leaves Important Questions Unanswered

**DOI:** 10.1097/HS9.0000000000000052

**Published:** 2018-06-11

**Authors:** Peter Borchmann, Alexander Fosså, Monika Długosz-Danecka, Boris Böll, Markus Dietlein, Carsten Kobe, Helen Goergen, Andreas Engert

**Affiliations:** 1 1st Department of Internal Medicine, University Hospital of Cologne, Cologne, Germany; 2 German Hodgkin Study Group, University Hospital of Cologne, Cologne, Germany; 3 Department of Medical Oncology, Oslo University Hospital, Oslo, Norway; 4 Department of Hematology, Jagiellonian University, Krakow, Poland; 5 Department of Nuclear Medicine, University Hospital of Cologne, Cologne, Germany

In 2010, the *New England Journal of Medicine* (*NEJM*) published phase I results of brentuximab vedotin (BV), an antibody-drug conjugate targeting CD30.^1^ After decades of standstill in the commercial development of new drugs in Hodgkin lymphoma (HL), this pioneering innovation opened up new options in this malignancy affecting mainly young adults. Phase II results of BV in relapsed or refractory (r/r) classical HL (cHL) then revealed an exceptional overall response rate of 75%, which led to approval.^2^ This was the first study reporting only positron emission tomography (PET)-based response rates, which were not comparable to results from all previously published studies using computed tomography (CT)-based response assessment. The more relevant progression-free survival (PFS) was less impressive with a median of 5.6 months. Subsequently, BV has been widely used as “bridge-to-transplant” for r/r HL patients. A phase 3 study then proved the activity of single-agent BV as consolidation after high-dose chemotherapy.^3^ In this setting, cHL patients treated with BV for 1 year had a significantly better outcome than patients in the placebo control arm. Accordingly, BV has been approved for relapsed cHL patients based on trials without any active comparator.

Nonetheless, the idea of using a targeted drug with high efficacy and good tolerability for cHL is attractive and BV might also be a promising drug to improve the treatment of newly diagnosed cHL. This is especially true for patients with advanced-stage disease. Here, 6 to 8 cycles of ABVD (doxorubicin, bleomycin, vinblastine and dacarbazine) used to be the standard of care in many countries. ABVD can be safely administered in the outpatient setting and is rather inexpensive. However, this regimen clearly needs improvement in terms of efficacy. This was and still is attempted either by early PET-based selection of poor responders and subsequent treatment intensification or by introducing new drugs.^4^ The respective phase II trial combined BV with ABVD or AVD to set the ground for a phase III study. Unfortunately, 2 patients in this trial treated with BV plus ABVD died due to lung toxicity of the combination of BV and bleomycin.^5^ The sponsor then decided to test the regimen without bleomycin in the BV + AVD regimen (BV, doxorubicin, vinblastine, and dacarbazine). Lung toxicity was less pronounced and this regimen was selected to challenge ABVD in the ECHELON-1 prospectively randomized phase III trial.^6^

It is important to remember that the development of the BV + A(B)VD regimen was driven by the idea to challenge ABVD in an add-on design, which obviously could have been an elegant way to show its efficacy in a company-sponsored pivotal study. Unfortunately, the active compound of BV, monomethyl auristatin E (MMAE), is a microtubule inhibitor, similar to vinblastine being a pivotal part of the A(B)VD regimen. Microtubule inhibitors usually have a rather narrow therapeutic window, which is especially true for MMAE. Oncologists usually do not use higher doses of microtubule inhibitors or combine 2 microtubule inhibitors. This is because one should expect a steep increase of dose-dependent toxicity, especially neutropenia and neuropathy without a comparable increase in efficacy. Despite such considerations, the combination of BV (MMAE) and vinblastine in the BV-AVD regimen was tested in the experimental arm of the ECHELON-1 study.

The *NEJM* published the ECHELON-1 data by Connors and colleagues and the results were also shown in the plenary session of the 2017 ASH meeting.^6,7^ The authors reported a total of 1334 patients with previously untreated stage III or IV cHL, of whom 664 were assigned to BV + AVD and 670 to ABVD. The primary endpoint was modified progression-free survival (mPFS), defined as the time to progression, death, or noncomplete response and use of subsequent anticancer therapy as adjudicated by an independent review committee. Two-year mPFS rates in the BV + AVD and ABVD groups were 82.1% (95% confidence interval [CI], 78.7–85.0) and 77.2% (95% CI, 73.7–80.4), respectively, with a difference of 4.9% points (hazard ratio [HR] 0.77; 95% CI for HR, 0.60–0.98; the originally published *P* value = 0.03 was corrected to 0.04 in an erratum published in March 2018, *N Engl J Med* 2018; 378:878). Based on the significant difference, the authors concluded that BV + AVD “had superior efficacy to ABVD in the treatment of patients with advanced-stage Hodgkin lymphoma.”

Many questions arise with the publication of this company-sponsored study in patients with newly diagnosed cHL. First, the primary endpoint mPFS introduced in this study has not been established in clinical research. In contrast to the well-established endpoint PFS, which evaluates time to disease progression or death, mPFS is per definition subject to bias, counting optional additional anticancer treatment including radiotherapy as a failure event. ECHELON-1 was an unblinded study in that physicians knew whether their patients received ABVD or the new BV + AVD treatment. In case of non-CR at the end of treatment, this could have impacted the decision on whether additional therapy was needed. These considerations clearly reflect the vulnerability of mPFS to bias indicating the weakness of this endpoint. The medical community relies on relevant established endpoints to put results of clinical trials such as ECHELON-1 into perspective. So far, this information has not been delivered. Looking at the numbers reported in the manuscript, the difference in terms of mPFS seems moderate (117 vs 146 events for BV + AVD and ABVD, respectively). A considerable proportion of mPFS events are in the category “noncomplete response and use of subsequent anticancer therapy as adjudicated by an independent review committee” (9 vs 22, respectively), and only 2 and 4 of these mPFS events were confirmed as PFS events by the independent review committee (see Table 1). It thus seems likely that the actual PFS difference is smaller than the mPFS difference and might even miss statistical significance.

**Table 1:**
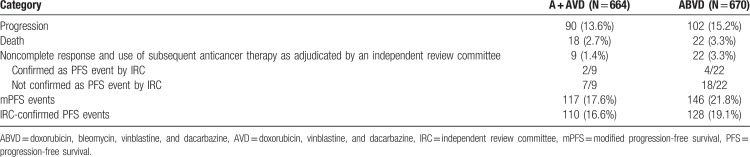
mPFS and IRC-Confirmed PFS Events in ECHELON-1

In addition, other questions regarding the primary endpoint in the ECHELON-1 trial should be addressed: the study was originally designed to show a 7.5% difference (82.5% vs 75%, HR 0.67) in favor of the BV + AVD regimen at 3 years with 1040 patients. However, the study was amended in order to evaluate the primary endpoint at 2 years already. According to this amendment, 1240 patients would have been needed to show a mPFS difference of 8% (HR 0.67), assuming a mPFS of 73% at 2 years for ABVD, which is less than initially assumed for 3 years. Finally, 1334 patients were evaluable for the primary endpoint. Thus, the study was obviously over-recruited and the relevance of the *P* value should be judged with caution. We can safely conclude that the difference was smaller than expected (observed 4.9%, expected 8%; HR observed 0.77, HR expected 0.67).

Therefore, we should discuss whether the moderate *statistically significant* benefit in terms of mPFS at 2 years is actually *clinically relevant*. This depends critically on 2 major parameters: the long-term outcome, which we obviously do not know yet, and the risk-to-benefit ratio of the new regimen, which the authors did not discuss adequately.

When observing a moderate benefit for a vulnerable primary endpoint, the experimental regimen could still be beneficial for the patient if it was better tolerated. However, the opposite is true for ECHELON-1: with the exception of severe lung toxicity, which occurred at rather low levels with both regimens (<1% vs 3% for BV + AVD vs ABVD, respectively); all other reported adverse events (AEs) were clearly more frequent with BV + AVD. This is true for severe toxicities ≥ grade 3 (83% vs 66%), serious AEs (43% vs 27%), hospitalizations (37% vs 28%), and others. In particular, severe neutropenia and severe neuropathy occurred more often with BV + AVD containing 2 microtubule inhibitors. This raises doubts on the usefulness of the experimental regimen. Grade 2 or higher peripheral neuropathy can be a disabling AE, which should be particularly avoided in these young HL patients. Overall, 30% of BV + AVD-treated patients suffered from neurotoxicity of at least grade 2. Unfortunately, the authors did not report on the persistence of neuropathy. We can only speculate how well neuropathy will resolve to a clinically acceptable degree. Clearly, the community needs exact numbers on frequency and severity of neuropathy to be reported at 2 years. All these numbers suggest that the risk-to-benefit ratio for BV + AVD is not convincing. We strongly believe that treatment-related risks should be taken into account,^8^ especially when there is no convincingly demonstrated benefit in terms of relevant endpoints such as PFS or overall survival.

Regarding the disadvantage of BV + AVD in terms of tolerability, the presented information (or better, the lack of such) and the corresponding conclusions also raise concern. For example, the authors conclude that BV + AVD is “a safe and at least equivalently effective treatment for older patients compared to ABVD.” However, older patients did not benefit from BV + AVD at all (HR = 1.0 [0.6–1.7]). Since neuropathy and neutropenia are usually more common and pronounced in elderly patients, there might be a particular risk in this cohort receiving treatment with BV + AVD. The authors did not report data that would either support or disprove this part of their conclusion. However, the frequency of CTCAE grade 3 to 4 neutropenia in BV + ABVD arm should be mentioned at this point—it occurred originally in 73% of the patients and led to protocol amendment implementing primary mandatory G-CSF prophylaxis. Another example of conclusions made without presentation of relevant data is the discussion of neurotoxicity, stating that the BV + AVD regimen “is associated with more […] neurotoxicity (which is largely reversible) than ABVD but substantially less pulmonary toxicity.” While valid data on reversibility of neurotoxicity are not presented in the manuscript as described above, a substantial decrease of severe pulmonary toxicity can hardly be stated given the low level of 3% for ABVD in this trial. Taken together, the risk-to-benefit ratio of this new regimen cannot be discussed without sound data.

Finally, the presentation of study results ignores the current standard of care, which is individualized, response-adapted therapy guided by early PET. The authors do not even mention the RATHL trial in their discussion, which has recently established PET-guided ABVD in the treatment of advanced-stage HL. The disregard of current standards also includes PET-guided eBEACOPP (dose-intensive courses of bleomycin, etoposide, doxorubicin, vincristine, procarbazine, and prednisone), which is used as standard of care in many European countries and is clearly more effective than BV + AVD (see Table 2).^9^

**Table 2:**
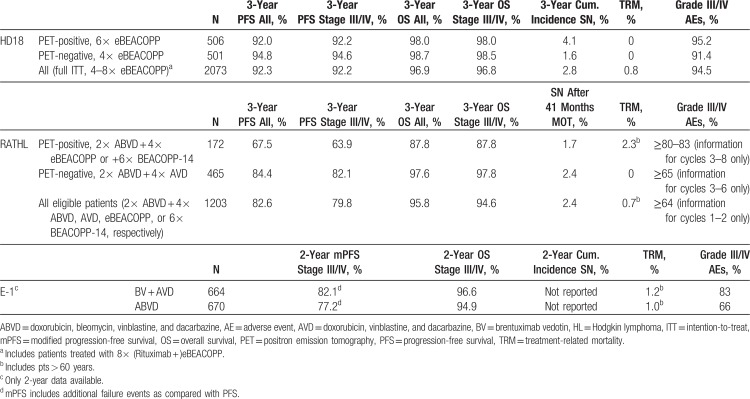
Outcome Data of Standard-of-Care-Defining Studies in Advanced-Stage HL

In summary, the ECHELON-1 trial raises concern for many reasons. First, the primary endpoint mPFS is subject to bias per definition. This problem could be overcome by presenting PFS data. Reported numbers suggest that there is no relevant PFS difference, but full data are needed to judge the potential benefit of the new regimen. Second, taking into account the marginal increase in efficacy, the obvious increase of toxicity needs to be presented in detail in order to balance risks and benefits of the BV + AVD regimen. Third, treatment of HL has evolved while the ECHELON-1 study was recruiting. Today, response-adapted therapy based on early PET-CT has become standard of care and allows tailoring the treatment intensity to the individual patient's need. Exposing 100% of patients to a treatment with clearly more AEs in order to achieve a minor (<5%) and questionable (mPFS) benefit seems to be an outdated strategy. Finally, the disregard of current standards in the presentation and discussion of ECHELON-1 does not support the academic discourse, which is essential to find the best way to treat our patients. The history and success of academic clinical research in HL reflects the huge potential of this academic discourse.
